# Risks and interrelationships of subdistrict house prices: the case of Amsterdam

**DOI:** 10.1007/s10901-017-9568-z

**Published:** 2017-08-21

**Authors:** Alfred Larm Teye, Jan de Haan, Marja G. Elsinga

**Affiliations:** 1 Department of Research for the Built Environment (OTB), Faculty of Architecture and the Built Environment, Julianalaan 134, 2628 BL Delft, South-Holland Netherlands; 2 Division of Corporate Services, IT and Methodology, Statistics Netherlands (CBS), Henri Faasdreef 312, 2492 JP The Hague, Netherlands

**Keywords:** Hedonic index, House prices, Lead–lag effect, Property price risk, Subdistricts, Amsterdam

## Abstract

This paper uses individual house transaction data from 1995 to 2014 in Amsterdam to explore the risks and interrelationships of the subdistrict house prices. Simple indicators suggest that house prices grow faster and are more risky in the central business district and its immediate surrounding areas than in the peripherals. Furthermore, we observe an over time decreasing intervariations between the subdistrict house price growth rates, whereas we find a lead–lag and house price causal flow from the more central to the peripheral subdistricts.

## Introduction

House price developments have significant wealth effect on households because of the large outlays involved in residential property investments. In 2009, Statistics Netherlands (CBS) estimated a total of 738,449 million euros wealth in residential properties for the Netherlands. By 2012, however, the total wealth had declined to 721,018 million euros (2.36%), showing a considerable amount of financial risks involved in residential property investment. Such risks are inherent in the dynamics of house prices, which need a better understanding particularly after the 2007–2008 Global Financial Crisis (GFC).

In this paper, the aim is to compute indicators that characterise the risks of residential house prices specifically at the lower-level districts and to study the interrelationships between these subdistrict house prices. While the price risks reveal unique characteristics of the house price development in each subdistrict, the interrelationships show how the house price development in a subdistrict is connected to the growth in the other subdistricts. These analyses at the lower-level districts may unveil important residential asset wealth distribution that is not available at the aggregate national or provincial level. Such information may be of interest to stakeholders, including statistical agencies, households, institutional investors and policy makers who control the overall functioning of the city-wide housing market. We obtain dataset for individual house transactions between 1995 and 2014, which enables us to analyse the case of the city of Amsterdam.

The residential property market of Amsterdam, which is also the capital city, is an interesting case to study in the Netherlands. Residential properties are usually more expensive in Amsterdam than in the other cities, which may be due to the higher demand for the capital where many employment opportunities and social amenities exist. Over time, the development pattern of Amsterdam house prices also differs considerably from other locations. Following the GFC, for example, house prices in Amsterdam declined more sharply but also recovered quicker than in other major Dutch cities, such as The Hague, Rotterdam and Utrecht.

To begin the analysis, customised house price indexes are created for the lower-level districts using the time-dummy hedonic method. We next estimate simple statistics from the indexes to characterise and to compare the risks of house prices in the subdistricts. Finally, we study two aspects of the interrelationships between the house prices: (1) the intervariation between the subdistrict house price returns (or growth rates), and (2) the lead–lag relationships between the subdistrict house prices.

The paper adopts risk metrics that include specifically the standard deviation, semi-deviation, and the ‘decline severity’. The standard deviation is a measure of the dispersion of the temporal (period-to-period) house price growth rates from the average, while the semi-deviation is a version of the standard deviation that considers the average deviation of only values below the mean. The semi-deviation is one of the commonly used downside risk measures for investment analysis in the mainstream finance literature, but it is surprisingly applied seldom in the housing context (see Wolski 2013; Foo and Eng 2000; Grootveld and Hallerbach 1999). The ‘decline severity’ is similar to the semi-deviation but captures the variation of returns which actually fall below zero.

The lead–lag relationships between the subdistrict house prices are studied using the Granger causality technique, while a version of the semi-deviation, which we refer to in this paper as the ‘interdistrict deviation,’ is used to study the intervariation between the growth rates. The interdistrict deviation is defined as the variation of the annual house price growth rate in one subdistrict from the growth rate across all the subdistricts. In the course of life, Dutch households usually purchase a property in a less desirable location with the intention of moving to a more desirable area when there is increase in disposable income (Banks et al. 2015; Droes et al. 2010; Sinai and Souleles 2003). This tendency, however, could be affected by the extent of variations in the growth of house prices across the various locations. The interdistrict deviation captures these locational house price differences.

The rest of the paper is structured as follows. The method and construction of the metrics are specified in Sect. 3, following a brief overview of the literature in Sect.  2. The data are described in Sect. 4. Section 5 discusses the empirical estimates of the metrics and analyses the interrelationships between the subdistrict house prices. Section 6 summarises the results and concludes the entire paper.

## Overview of the literature

This paper focuses mainly on residential property price risks and the interrelationship between the house price developments. The property price risk is here referred to as the potential loss on investment in residential properties due to a fall in property prices. It is important to study this risk because changes in house prices tend to affect the balance sheet of households and other significant parts of the economy (Dolde and Tirtiroglu 2002; Duca et al. 2010). The 2007–2008 GFC especially has lent some credence to the notion that stress in the financial sector may ensue from collapse in real estate prices (Aalbers 2009; Baker 2008).

Many authors use the volatility defined by the standard deviation to measure the property price risk in the literature (e.g. Ross and Zisler 1991; Miller and Pandher 2008; Dolde and Tirtiroglu 2002). However, it is well known that this measure accounts only for the variations in the house price distribution from the average and does not necessarily capture the downside risk, which would be preferable. Jin and Ziobrowski (2011) proposed using the value-at-risk (VaR) instead of the standard deviation. This measure is a downside risk metric that indicates the worst-case loss on a portfolio held over a short period of time, given a certain confidence level (Crouhy et al. 2006).

Although widely used in the mainstream financial literature, many researchers criticise the VaR for violating certain mathematical axioms, which, it is argued, disqualifies it from being a coherent risk measure (see Acerbi and Tasche 2002; Yamai and Yoshiba 2002; Szegö 2002).^1^ The metric is also known to be more sensitive to the underlying distribution of the price return. Where the returns are not normally distributed, for instance, it is observed that the VaR may inaccurately estimate losses, which may then tempt investors to choosing portfolios with risky profiles (Hull 2006).

This article aims to compare house price risks in smaller subdistrict markets using summary statistics. Simple summary statistics may be informative for the individual households and institutions that must make decision on housing investments in a particular subdistrict. We use three metrics (the standard deviation, semi-deviation and decline severity), which are based on localised price indexes constructed for each of the lower-level districts. The indexes are created with the time-dummy hedonic method (TDHM). The TDHM is a widely used approach that is based on the idea that house prices can be described by their physical and locational attributes (Rosen 1974; Malpezzi et al. 2003). Our dataset contains details on these physical and locational features which enable application of the TDHM in this paper.

The procedure for the TDHM mainly involves a regression of time-dummy variables and the characteristics on the logged property sale prices (see de Haan and Diewert 2013; Hill 2013). This regression equation can easily be estimated by the method of ordinary least squares (OLS), and the estimated coefficients could then be converted into a constant quality price indexes (time-dummy hedonic price indexes). The indexes uniquely reflect the development of house prices in each of the subdistricts. Nonetheless, significant interrelationships may also exist between these subdistrict house prices. For instance, due to economic activities, such as migration and equity transfer, shocks to property prices may spread from one location to the other places with a transitory or permanent impact (Meen 1999; Holly et al. 2011).

The phenomenon, in which house price shocks spread over their influence from one region to another, is often referred to as the ripple or spillover effect in the literature and was first observed by researchers in the UK (Giussani and Hadjimatheou 1991; MacDonald and Taylor 1993; Meen 1999). Later, research in other countries also supported the ripple effect hypothesis. Empirical studies by Berg (2002), for example, using second-hand family houses in Sweden found evidence supporting the ripple effect existing from Stockholm to other regions. In the USA, Canarella et al. (2012) investigated the spatial interrelationships between house prices and concluded on a ripple effect potentially existing from the east and west coast metropolitan areas to the rest of the USA. Helgers and  Buyst (2016), who investigated the case of Belgium, also found that house price shocks are more likely to spread from Antwerp to other parts of the country. Comparable results were found in China by Gong et al. (2016b) and for South Africa by Balcilar et al. (2013).

In the Netherlands, however, there is a dearth in the literature regarding the spatial interrelationships between house prices. This paper contributes to the subject by studying the lead–lag effect between the lower-level-district house prices of Amsterdam using the Granger causality technique. The concept of Granger causality (GC), popularised in the literature by Granger (1969), is one of the simple empirical methods that has been used widely for testing the lead–lag effect and the ripple effect between regional house prices. It is has been applied by, for example, Giussani and Hadjimatheou (1991) and recently by Gong et al. (2016b), who studied the ripple effect between regional house prices.

## Empirical method

A time-dummy hedonic house price index is first constructed for each subdistrict. Statistics Netherlands designate 15 subdistricts in Amsterdam for official statistical purposes, which are also adopted in this paper. Rosen (1974) defines hedonic prices as the “implicit prices of attributes that are revealed to economic agents from observed prices of differentiated products and the specific amounts of characteristics associated with them”. The time-dummy hedonic model (TDHM) includes the period of transaction as one of the characteristics, following the definition of Rosen (1974). In the notations of de Haan and Diewert (2013), the estimating regression equation of the TDHM could be described by the model:

1 $$\begin{aligned} \ln p^t_n = \beta _0 + \sum _{\tau =1}^{T}{\delta ^\tau D_n^{\tau }} + \sum _{k=1}^{K}{\beta _k z_{nk}^t} + \varepsilon _n^t \end{aligned}$$

where $p^t_n$ is the price of the nth property in the period *t* from the sample of $N_t$ properties with *K* number of characteristics $z^K=(z_{nk}^t)_{k=1}^K$. $\varepsilon _n^t$ is the error term assumed to be white noise process, whereas $D_n^{\tau }$ is the time dummy that takes the value one if $p^t_n$ belongs to the sample $N_t$ and zero otherwise. $T>1$ is the length of the sample period. By omitting one of the dummy variables (usually the base period), Eq. (1) is estimated on the pooled data by the method of OLS and the index tracking the growth rate from time 0 to $\tau$ is simply obtained with the exponentiation $\pi ^{\tau } = \exp (\hat{\delta }^\tau )$. Here, $\hat{\delta }^\tau$ denotes the estimate of $\delta ^{\tau }$.

### Risk indicators

For each of the subdistrict (*i* say), we follow the above procedure to estimate the house price index from 1995 to 2014, using 1995 as the base year. After that, the standard deviation and the semi-deviation measuring the house price risks are constructed as the square root of the quantities, $\sigma _i^2$ and $\gamma _i^2,$ respectively, defined by;

2 $$\begin{aligned} \sigma _i^2&= (T-1)^{-1}\sum _{t=1}^{T}{\left( d^i_t-\mu _i\right) ^2} \nonumber \\ \gamma _{i}^2&= (T-1)^{-1}\sum _{t=1}^{T}{\left( \min (d^i_t-\mu _i,0)\right) ^2} \end{aligned}$$

where $\mu ^i = T^{-1}\sum _{t=1}^{T}{d^i_t}$ is the mean house price return in the subdistrict *i*. The (temporal) house price returns are defined as $d^i_t=\pi ^t_i/\pi ^{t-1}_i -1$. The semi-deviation considers only the returns below the mean, which makes it a downside risk metric that has a more appealing connotation for risk than the standard deviation.^2^

Similarly, we define the ‘decline severity’ as the average over the growth rates that are actually below zero. This is specifically written as the square root of $\delta _i^2$, where

3 $$\begin{aligned} \delta _{i}^2= (T-1)^{-1}\sum _{t=1}^{T}{\left( \min (d^i_t,0)\right) ^2} \end{aligned}$$

Because $\delta _i^2$ considers only the returns below zero, the ‘decline severity’ may accurately capture the true losses than the semi-deviation which includes returns below the mean that do not necessarily represent losses.

### Subdistrict house price interrelationships

Two aspects of the interrelationships between subdistrict house prices (the intervariation between growth rates and the lead–lag effects) are considered in this paper. We study the intervariation between the subdistrict house price growth rates, using the “interdistrict” deviation. The interdistrict deviation gives indication of how far house prices in a particular subdistrict are growing below the rates in the other subdistricts. It is expressly defined as the square root of $\phi _{i}^2$, where

4 $$\begin{aligned} \phi _{i}^2&= [(L-1)(T-1)]^{-1}\mathop{\mathop{\sum}\limits_{j=1}}\limits_{j\ne i }^{L-1}{\sum _{t=1}^{T}\left( \min (d_t^i-d^j_t,0)\right) ^2} \end{aligned}$$

$L>1$ is the total number of subdistricts. The definition of $\phi _{i}^2$ is a version of the semi-variance statistically expressed as the squared deviations of the house price growth rates $d_t^j$ in the subdistricts *j* that fall above the rate $d^i_t$ in the district *i*. It may be considered as the premium for a house move within the municipality. For housing-related government compensation of a sort, the interdistrict deviation may also give indication of the discrepancy between the housing worth of households which would determine the benefit in each subdistricts.

To study the lead–lag effects between the growth rate of subdistrict house prices, the pairwise Granger causality (GC) method is adopted. Let $x^i_t$ and $x^j_t$ be the growth rates from the respective subdistricts *i* and *j*. The empirical procedure for the pairwise GC test is to first estimate the regression equations:

5 $$\begin{aligned} x^i_t = \alpha _0 +\sum _{k=1}^p{\alpha _{1k}x^i_{t-k}}+\sum _{k=1}^p{\beta _{1k}x^j_{t-k}}+\epsilon _{1t} \nonumber \\ x^j_t = \beta _0 +\sum _{k=1}^p{\alpha _{2k}x^j_{t-k}}+\sum _{k=1}^p{\beta _{2k}x^i_{t-k}}+\epsilon _{2t} \end{aligned}$$

where $\epsilon _{1t}$ and $\epsilon _{2t}$ are uncorrelated disturbance terms. The lag *p* may be determined with an information criterion (AIC or BIC). Formally, $x^j_t$ Granger causes $x^i_t$ if the estimated parameters $\beta _{11},\ldots ,\beta _{1p}$ are statistically different from zero. That is, $x^j_t$ Granger causes $x^i_t$ if the hypothesis $H_0^i: \beta _{11}=\cdots =\beta _{1p}=0$ is rejected at a reasonable statistical significant level. Similarly, $x^i_t$ Granger causes $x^j_t$ if we can reject the hypothesis $H_0^j: \beta _{21}=\cdots =\beta _{2p}=0$ at a reasonable statistical significant level.

## Description of data

The analysis in this paper uses dataset on individual sale transactions in Amsterdam between 1983 and 2014. This dataset is obtained from the realtor organisation NVM.^3^ Information on about 150,000 transactions was received in total. The NVM’s coverage of sales information in the Netherlands has been improving over the years. The average coverage per year is generally about 75%. However, we discovered that the NVM data had no information on the dwelling characteristics for a large portion of the sales reported prior to 1995. Since these records are needed to construct the time-dummy hedonic indexes, all observations before 1995 were discarded.

For the rest of the dataset, we sought to construct house price indexes for existing dwellings and we therefore removed newly build homes, which totalled 4169. A more detailed data cleaning was carried out following Diewert (2010), who estimated various hedonic house prices indexes using similar dataset. Specifically, observations with missing transaction prices (these are set to −1 by the NVM) and those with unusual values (e.g. 0s, 9s) were excluded. We also omitted observations with recorded transaction prices in excess of €4 million (74), and those below €10,000 (404).

The records with extremely small house sizes^4^ (below 20 m$^2$) in addition to the observations with unavailable structure sizes (3642 in total) were excluded as well. Furthermore, we deleted five observations for which the property type was unavailable or unknown. The remaining data, constituting a total sample size of 116,446, were finally divided into the 15 statistical subdistricts of Amsterdam.

**Fig. 1:**
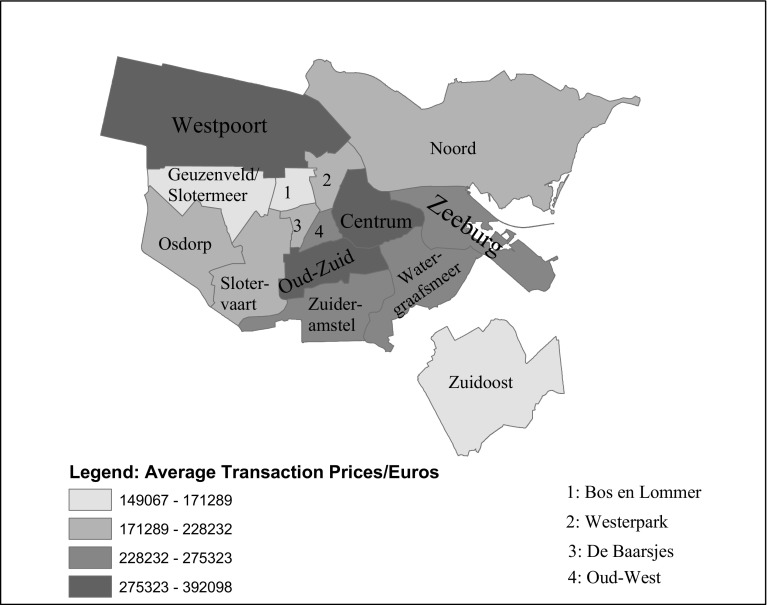
Local districts and neighbourhoods in the city of Amsterdam. Average transaction prices are based on NVM data from 1995 to 2014. *Source* CBS, NVM

Figure 1 and Table 1 present the summary statistics for the remaining data. A brief look at the figure and the table indicates that during the study period, houses in Amsterdam sold for an average of about €261,513. Average house prices in less expensive areas like Zuid-Oost, Geuzenveld en Slotermeer, Bos en Lommer and Noord were below €200,000. The more expensive districts include the central business district (Centrum) and its immediate surroundings (Westpoort and Oud-Zuid), where average price were above €300,000. In addition to the locational attributes, there is significant disparity in the average disposable income of local residents, which may contribute to house price variations between the subdistricts (see Amsterdam 2013).

**Table 1:** Summary statistics for transactions from 1995 to 2014

Subdistrict	Total observations	Mean price (euros)	Standard deviation	Average usable area (*m* $^2$)	Average age (decades)
Centrum	16,805	344,293.0	238,061.9	97.0	5.85
Westpoort	0041	392,098.4	174,284.3	87.8	0.54
Westerpark	5958	228,231.9	126,395.0	69.9	5.75
Oud-West	7633	275,323.4	184,124.0	80.4	6.79
Zeeburg	7628	266,334.1	142,666.7	88.7	2.80
Bos en Lommer	5009	171,289.3	81,045.08	69.0	5.87
De Baarsjes	6547	202,730.7	102,998.6	71.8	6.52
Noord	8521	193,182.5	111,130.2	89.9	3.94
Geuzenveld en Slotermeer	3720	164,187.6	79,909.1	83.7	3.62
Osdorp	5518	194,725.1	110,606.0	97.6	2.63
Slotervaart en Overtoomse Veld	4565	225,467.8	123,070.2	101.0	2.20
Zuid-Oost	6842	149,067.1	72,615.4	86.3	2.33
Watergraafsmeer	8409	258,422.4	142,885.8	87.2	5.46
Oud-Zuid	18,830	348,942.8	278,432.5	96.8	6.73
Zuideramstel	10,420	272,807.0	185,531.9	93.8	5.07
Whole of Amsterdam	116,446	261,512.6	193,972.7	88.9	5.07

*Source*: Authors’ computations based on NVM data

The larger population also significantly affects house price developments in Amsterdam.^5^ In 2013, for example, there was a housing deficit of almost 31,370 due to the larger number of households. The estimated number of households was about 431,370, while the total housing stock stood at about 400,000 in 2013 (Amsterdam 2013). The housing deficit in Amsterdam is generally persistent and eventually has a considerable impact on house prices (see Dröes and Van de Minne 2015; Minne et al. 2015).

## Empirical estimation and results

### Subdistrict indexes

The localised house price indexes were constructed for 15 of the Amsterdam subdistricts using the TDHM. Westpoort was omitted because there were only few observations which did not cover the entire study period.^6^ The implementation of the TDHM first requires that choice be made about which dwelling characteristics to include in the regression Eq. (1). We begin with several characteristics and then exclude those features that were statistically insignificant across the fourteen districts using the *p* values. The final regression uses the log transaction prices as dependent variable and only seven explanatory variables, most of which are categorised into the several groups described in Table 4.

Including the time dummies (the base period 1995 omitted for identifiability of the model), the adjusted R-squared showing the proportion of variation in log transaction prices explained across the 14 districts ranges from 80.33% to about 90.41%. The same factors in addition to the location (district) dummies indicating the districts of transaction explain nearly 84.24% of the variation in log sale prices across the whole Amsterdam. The regression result for the entire Amsterdam is presented in Table 5.

It is noticeable that the estimated coefficients of most of the explanatory variables are statistically significant (even at the 1% level) and that they also carry the expected signs. More specifically, the coefficients of the total usable area, the number of rooms and the number of floors are all positive and statistically significant. The location of the house and the property type also play an important role in determining the property prices, as expected. Compared to the central district (Centrum), the regression results show that prices are lower in all other districts except in Westpoort. The maintenance level inside the property also has a positive impact on the price of the property. We note, however, that the maintenance level compiled by the NVM is rather more subjective to the property valuer during the transaction.

The age coefficient is negatively signed, which might appear counter-intuitive at first sight. However, older dwellings tend to be more expensive because many Dutch people prefer them, especially when they are located along monumental streets and close to museums or other public areas. A further look at Table 1 and Fig. 1 indeed reveals that except Westpoort, most of the subdistricts closer to the central area of the city where properties are more expensive also have comparatively older dwellings.

**Fig. 2:**
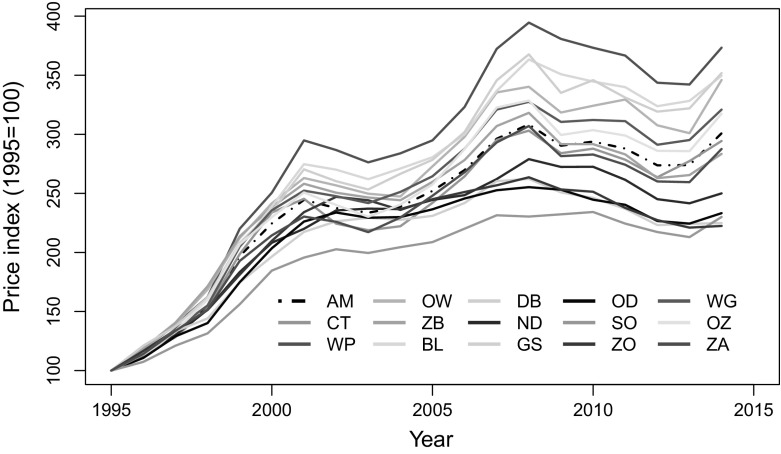
The city-wide Amsterdam and the local residential property prices indexes compared. *AM* Amsterdam, *CT* Centrum, *WP* Westerpark, *OW* Oud-West, *ZB* Zeeburg, *BL* Bos en Lommer, *DB* De Baarsjes, *ND* Noord, *GS* Geuzenveld en Slotermeer, *OD* Osdorp, *SO* Slotervaart en Overtoomse Veld, *ZO* Zuid-Oost, *WG* Watergraafsmeer, *OZ* Oud-Zuid, *ZA* Zuideramstel. *Source* Author’s estimate from NVM data

The house prices indexes are constructed by the exponentiation of the estimated year dummy coefficients as described in Sect. 3. Figure 2 compares the indexes from the 14 districts with the city-wide Amsterdam price index. The plot reveals significant differences in the house price developments across the Amsterdam subdistricts. Compared to the city-wide trend, house prices are generally higher and more volatile in Westerpark, Oud-West, Bos en Lommer and De Baarsjes. A few of the subdistricts (Centrum, Zeeburg and Zuidamstel) closely mimic the city-wide house price trend especially after 2005, whereas subdistricts, such as Slotervaart en Overtoomse Veld, Osdorp, Geuzenveld en Slotermeer and Zuid-Oost, that are on peripheral have lower and more stable house prices. As in Fig. 1, it is observable here too that those subdistricts that are closer to the city centre tend to have higher house prices over time.

### House price returns and risks

**Fig. 3:**
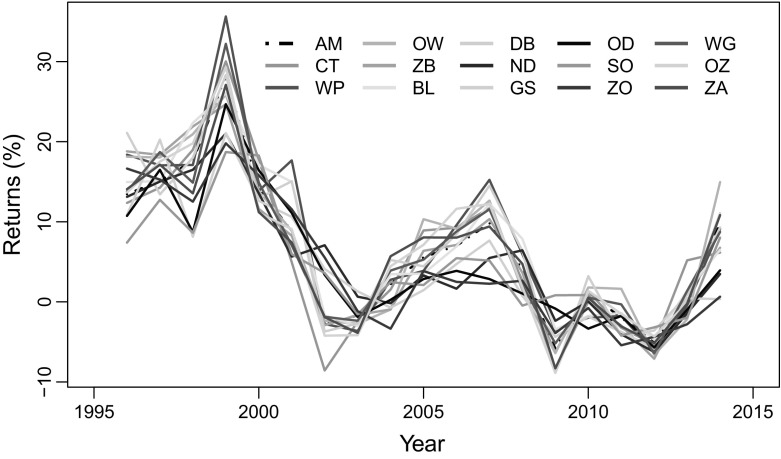
Temporal house price returns. *AM* Amsterdam, *CT* Centrum, *WP* Westerpark, *OW* Oud-West, *ZB* Zeeburg, *BL* Bos en Lommer, *DB* De Baarsjes, *ND* Noord, *GS* Geuzenveld en Slotermeer, *OD* Osdorp, *SO* Slotervaart en Overtoomse Veld, *ZO* Zuid-Oost, *WG* Watergraafsmeer, *OZ* Oud-Zuid, *ZA* Zuideramstel *Source* Author’s estimate from NVM data

This subsection reports on the returns and risks of house price for the subdistricts. The temporal returns ($d^i_t$) are displayed in Fig. 3. The risk measures here include the standard deviation, the semi-deviation and the decline severity, which are first computed aggregately over the entire study period and then over a rolling window of five years to discern the risk development pattern over time.

The aggregate result displayed in Table 2 shows that the annual house price growth rate is higher (greater than 7%) in Westerpark, Oud-West, Bos en Lommer and De Baarsjes, while this is relatively lower (less than 5%) in Osdorp, Zuid-Oost, Slotervaart en O. Veld and Geuz. en Slotermeer. Similarly, the standard deviation, semi-deviation and the decline severity all suggest that houses prices are of higher risk in Westerpark, Oud-West, De Baarsjes, Oud-zuid, centrum and Zeeburg than in the other subdistricts, which are more on the peripheral of the city.

**Table 2:** Average returns and risks of subdistrict house prices (1995 to 2014)

Subdistrict	Average return	Standard deviation	Semi-deviation	Decline severity	Rank of riskiness
Centrum	6.2686	9.8478	6.2498	**2**.**8847**	5
Westerpark	**7**.**6770**	**10**.**852**	**6**.**6735**	2.0471	1
Oud-West	7.1739	9.8267	6.5296	2.3352	2
Zeeburg	6.0465	9.6737	6.1424	2.7209	6
Bos en Lommer	7.1811	9.2690	5.8561	1.6393	7
De Baarsjes	7.2679	9.8317	6.4933	2.6208	3
Noord	5.1919	7.5457	4.6599	1.8257	11
Geuzenveld en Slotermeer	4.6212	7.7383	4.6024	1.9330	12
Osdorp	4.8312	7.9343	4.4694	1.6561	13
Slotervaart en Overtoomse Veld	4.6719	6.5636	3.9181	1.3419	14
Zuid-Oost	4.5900	8.1308	4.9299	2.1108	10
Watergraafsmeer	6.7140	9.5101	5.7046	2.0178	9
Oud-Zuid	6.6843	9.7729	6.3639	2.6630	4
Zuideramstel	6.0611	8.8373	5.8506	2.5516	8
Whole of Amsterdam	6.3069	8.8324	5.5124	1.9649	–

Mean return and risk figures are in percentages, with the maximum indicated in bold. The ranking is according to the semi-deviation

**Fig. 4:**
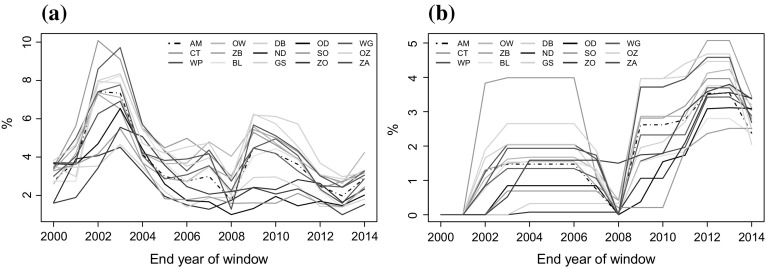
Pattern of subdistrict house price risk over time using a 5-year rolling window. **a** Semi-deviation, **b** Decline severity. *AM* Amsterdam, *CT* Centrum, *WP* Westerpark, *OW* Oud-West, *ZB* Zeeburg, *BL* Bos en Lommer, *DB* De Baarsjes, *ND* Noord, *GS* Geuzenveld en Slotermeer, *OD* Osdorp, *SO* Slotervaart en Overtoomse Veld, *ZO* Zuid-Oost, *WG* Watergraafsmeer, *OZ* Oud-Zuid, *ZA* Zuideramstel

Figure 4 displays the subdistrict risk developments overtime. The figure shows significant differences in the risk level between the subdistricts. The pattern overtime, however, do not vary much. For all subdistricts, the semi-deviation shows that house prices risk increases from 1995 until 2003 after which it became fairly stable. The decline severity, on the other hand, indicates that the house price risk was relatively stable for all subdistricts but increased sharply after 2008.

In 2007–2008, the GFC had a dramatic and negative impact on house prices and this is captured well by the decline severity measure. Following the crisis, house prices fell in Amsterdam by almost 12.56% between 2008 and 2013 (see Figs. 2, 3). Figure 4b, however, shows that the impact of the GFC varied significantly across the Amsterdam subdistricts. The impact appears severer especially in Oud-zuid, Oud-West, Zuideramstel, centrum and De Baarsjes, where house price returns below zero is higher between 2008 and 2103 (Fig. 4b). Although the semi-deviation and decline severity tend to have comparable risk values after 2008, the decline severity may be more accurate because it actually considers returns which are below zero. The semi-deviation, on the other hand, uses values below the average return that in principle may not indicate actual losses.

### Subdistrict house price interrelationships

### Intervariation

**Fig. 5:**
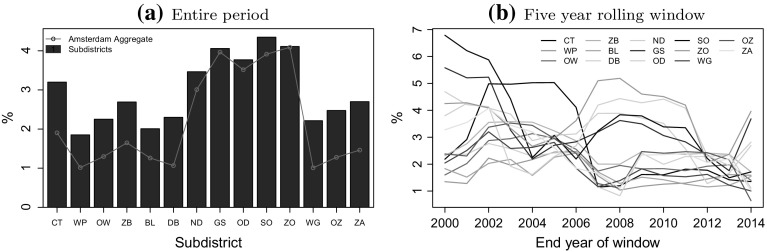
Amsterdam intersubdistrict house price deviations. **a** Entire period, **b** Five year rolling window. *CT* Centrum, *WP* Westerpark, *OW* Oud-West, *ZB* Zeeburg, *BL* Bos en Lommer, *DB* De Baarsjes, *ND* Noord, *GS* Geuzenveld en Slotermeer, *OD* Osdorp, *SO* Slotervaart en Overtoomse Veld, *ZO* Zuid-Oost, *WG* Watergraafsmeer, *OZ* Oud-Zuid, *ZA* Zuideramstel

The intervariation is use to mean the extent to which a particular subdistrict house price growth (or return) fall below the city-wide values. The intercity deviation (Eq. 4) is used to quantify the intervariations. The metric is computed first using the average of the indicated subdistrict deviation below the Amsterdam aggregated city-wide return series and then using the average deviation below the individual temporal returns of all the subdistricts. The former is depicted in red line and the latter in the blue bars of Fig. 5a. The figure indicates that subdistricts, including Noord, Geuzenveld en Slotermeer, Osdorp, Slotervaart en Overtoomse Veld and Zuid-Oost, where house prices are lower (see Fig. 2) generally have larger variation of house price returns below the average. Similarly, Oud-West, De Baarsjes, Oud-Zuid and Watergraafsmeer, among other subdistricts, with relatively expensive houses tend to exhibit lower return deviation below the city-wide average. For most subdistricts, the pattern over time (Fig. 5b) shows a slightly decreasing trend before 2008, while there are no significant changes afterwards.

### Lead–lag effect

The subdistrict house price returns may also exhibit lead–lag effects, besides the significant intervariations that exit between them. The lead–lag effect is confirmed in this paper using the Granger causality (GC) approach. In implementing the GC test, it is important that the house price return series are statistically stationary. The commonly used ADF (Dickey and Fuller 1979) and KPSS (Kwiatkowski et al. 1992) tests both confirm that the house price return series are stationary at sufficient statistical significant levels (see Table 6).

Table 3 summarises the results of the pairwise GC test, where the null hypothesis is that the subdistricts on the row do not Granger cause those on the columns. At the 5% statistical significance level, the results show considerable lead–lag effects between the subdistricts, with growth of house prices in any subdistrict being Granger-caused by at least one other subdistrict prices. Westpark house price returns, for example, is Granger-caused by as many as nine other subdistricts. Geuzenveld en Slotermeer and Osdorp are equally Granger-caused by eight and seven other subdistricts, respectively.

The pattern of lead–lag effects appears spatially complicated with the Granger causality not necessarily existing between subdistricts that border each other. However, it is observable that the causal flow occurs most from the more central subdistricts and close environs, including Zeeburg, Centrum and Oud-Zuid. Chen et al. (2011) and Gong et al. (2016a) similarly found that house price lead–lag effect and causal flow occur predominantly from the central to the peripheral districts. Meen (1999) suggests this kind of house price spatial interrelationship might occur through socio-economic activities such as internal migration and equity transfer (see also Pollakowski and Ray 1997).

**Table 3:** Pairwise Granger causality test results

Subdistrict	CT	WP	OW	ZB	BL	DB	ND	GS	OD	SO	ZO	WG	OZ	ZA
CT		14.37(1) 0.000***	1.889(4) 0.177	1.192(4) 0.364	5.832(1) 0.022**	9.565(1) 0.004***	1.440(1) 0.240	3.649(4) 0.036**	8.302(1) 0.007***	1.918(4) 0.172	4.685(1) 0.039**	3.760(1) 0.062*	0.762(1) 0.390	3.243(1) 0.082*
WP	1.662(1) 0.207		0.224(2) 0.801	5.752(1) 0.023**	0.830(1) 0.370	0.010(1) 0.922	1.449(1) 0.238	1.509(4) 0.261	2.042(1) 0.163	1.569(4) 0.245	1.722(1) 0.199	2.808(4) 0.074*	3.433(2) 0.049	2.888(2) 0.075*
OW	0.570(4) 0.689	0.689(2) 0.017**		0.514(1) 0.479	1.859(2) 0.178	6.944(1) 0.013**`	2.796(1) 0.104	3.137(4) 0.055*	7.799(1) 0.009***	2.291(2) 0.123	4.369(1) 0.045**	3.149(1) 0.0861*	0.456(1) 0.505	2.042(1) 0.163
ZB	2.110(4) 0.142	25.74(1) 0.000***	3.541(1) 0.070*		10.00(1) 0.004***	14.34(1) 0.001***	8.057(1) 0.008***	12.98(1) 0.001***	17.42(1) 0.000***	0.888(4) 0.500	6.939(1) 0.013**	0.308(4) 0.867	2.145(1) 0.153	6.693(1) 0.015**
BL	0.398(1) 0.533	9.470(1) 0.004***	0.362(2) 0.700	2.689(1) 0.112		2.429(1) 0.130	3.148(1) 0.086*	1.716(4) 0.211	2.727(4) 0.080	1.452(4) 0.277	6.059(1) 0.020**	1.146(2) 0.335	0.396(1) 0.534	0.300(1) 0.588
DB	0.814(1) 0.374	2.213(1) 0.147	0.142(1) 0.709	4.213(1) 0.049**	0.008(1) 0.929		2.293(1) 0.140	4.587(1) 0.040**	6.319(1) 0.018**	5.086(1) 0.032**	2.309(1) 0.139	1.174(2) 0.326	3.376(2) 0.051*	4.318(2) 0.025**
ND	0.060(1) 0.808	3.068(1) 0.090*	0.006(1) 0.938	0.383(1) 0.541	0.866(1) 0.360	1.506(1) 0.229		5.552(1) 0.025**	8.180(1) 0.008**	6.798(1) 0.014**	1.295(1) 0.264	0.718(1) 0.403	0.018(1) 0.896	0.214(3) 0.885
GS	1.242(4) 0.345	6.490(4) 0.005***	1.593(4) 0.239	0.397(1) 0.534	7.669(4) 0.002***	0.751(1) 0.393	0.817(1) 0.373		0.011(1) 0.917	1.442(1) 0.239	0.008(1) 0.929	3.056(4) 0.060*	0.254(1) 0.618	2.787(4) 0.075*
OD	0.587(1) 0.450	0.091(1) 0.765	0.143(1) 0.708	0.643(1) 0.429	11.27(4) 0.000***	0.553(1) 0.463	0.009 0.927	1.002(1) 0.325		7.248(3) 0.002***	5.997(4) 0.007***	0.816(1) 0.374	0.454(1) 0.505	0.365(1) 0.550
SO	10.68(4) 0.000***	8.302(4) 0.002***	1.396(2) 0.267	5.084(4) 0.012**	30.38(4) 0.000***	2.046(1) 0.162	0.000 0.991	1.520(1) 0.227	3.546(3) 0.035**		3.067(4) 0.059*	4.409(4) 0.0201**	0.034(1) 0.854	0.003(1) 0.955
ZO	0.091(1) 0.764	1.290(1) 0.265	0.080(1) 0.779	0.426(1) 0.519	0.206(1) 0.653	0.605(1) 0.443	0.275(1) 0.604	10.87(1) 0.002***	0.581(4) 0.682	4.736(4) 0.016**		0.190(1) 0.666	0.009 0.926	0.002(1) 0.966
WG	0.102(1) 0.752	3.850(4) 0.031**	0.074(1) 0.788	1.198(4) 0.361	1.796(2) 0.188	4.091(2) 0.030**	1.003(1) 0.325	3.919(4) 0.029**	3.355(1) 0.077*	2.005(4) 0.158	2.956(1) 0.096		0.525(1) 0.474	0.148(1) 0.703
OZ	0.458(1) 0.503	9.413(2) 0.000***	0.752(1) 0.393	0.104(1) 0.749	4.026(1) 0.054*	6.962(2) 0.004***	3.618(1) 0.067	4.472(1) 0.043**	6.549(1) 0.016**	7.744(1) 0.009***	4.864(1) 0.035**	4.039(1) 0.054*		3.340(1) 0.078*
ZA	0.005(1) 0.960	5.858(2) 0.008***	0.157(1) 0.695	0.692(1) 0.412	1.371(1) 0.251	8.045(2) 0.002***	0.391(3) 0.760	8.309(4) 0.002***	3.991(1) 0.055*	6.031(1) 0.020**	2.361(1) 0.135	0.275(1) 0.604	0.921(1) 0.345	

The null hypothesis is that the subdistricts on the row do not Granger cause those on the columns. Test regression is estimated with intercept. The lag *p* is determined by BIC and indicated in parenthesis. The Wald statistics are reported with the *p* values reported under it. $$*$$, $$**$$ and $$***$$ denote statistical significance at the 10, 5 and 1% respectively

## Concluding remarks

The 2007–2008 Global Financial Crisis (GFC) has given greater impetus to research seeking understanding into the dynamics and risks of house prices. Using dataset from Amsterdam on individual house transactions, this paper has explored summary statistics to measure the house prices risks and investigated the interrelationships between the subdistrict house prices. The summary statistics adopted are, namely, the standard deviation, semi-deviation and the decline severity, which is a variant of the semi-deviation. The interrelationships considered include the intervariation between the subdistrict house price returns and the lead–lag effects, which are studied within the Granger causality framework.

The key observations and conclusions of the paper could be summarised as the following. (1) House prices are generally more expensive and grow faster at the more central subdistricts and the immediate surroundings than in the peripherals. (2) There is an over time decreasing trend in the intervariations between the subdistrict house price returns. The intervariations are especially higher before the GFC, while they are lower and fairly constant afterwards. (3) The lead–lag relationships and house price causal flow occur most from the central to the peripheral subdistricts and this is similar to earlier empirical results by Gong et al. (2016a) and Chen et al. (2011).

In application, the risk metrics used in this paper may be of interest to statistical agencies. The metrics reveal important trends that are consistent generally with the Dutch house price development cycles. The decline severity especially is promising as a publishable risk metric for the housing market. It measures the variation of the temporal house price returns that are actually below zero and seems to capture the higher property price risk after the GFC more accurately than the other indicators (see Fig. 4). The results of the paper also provide useful information for policy regulations and for housing investors. For housing-related government compensation, for example, the interdistrict deviation may indicate the discrepancy between the housing worth of households which would determine the benefit for households in each subdistricts. The results indicating the risk distributions across the subdistricts and the interrelationships between the subdistrict house prices may equally guide investors to choose desirable locations for their investments.

For further investigation, however, it might be insightful to consider other empirical methods and the application of a more complex economic model to investigate the interrelationships between the subdistrict house prices. As Meen (1999) suggests spatial interrelationship between house prices might occur through socio-economic activities, including internal migration. The internal migration dynamics may be considered explicitly in the economic model.

## Appendix

See Tables 4, 5, and 6.

**Table 4:** Definition of explanatory variables in the time-dummy hedonic model **Source:** Extract from NVM data

Characteristics	Description	Variable type	Measurement unit
M2	Total usable floor area in square metres	Continuous	Positive real number
NKAMERS	Number of rooms	Continuous	Positive integer
NVERDIEP	Number of floors	Continuous	Positive integer
AGE	The age of the building in decades	Continuous	Non-negative integer
VERW	System of heating	Categorical	$0,\ldots ,3$
ONBI	Maintenance level inside the property	Categorical	$1,\ldots ,9$
HOUSETYPE	Type of house	Categorical	$2,\ldots ,7$
LOC	The district in which property is located	Categorical	$0,\ldots ,14$

Type of heating system: no heating system, gas/stove heating, central boiler heating and air condition/solar heating. Maintenance level: bad, poor to moderate, moderate, moderate to reasonable, reasonable, reasonable to good, good, good to excellent and excellent. Properties classes: terraced house, town house, corner house, semi-detached house, detached house and apartment. The location of the properties was categorised into 15 as specified in Table 1

**Table 5:** Hedonic regression estimates for the whole of Amsterdam

Variable	Estimate	SE	t value	$Pr({>}|t|)$
Intercept	1.070e+01	1.540e−02	695.003	<2e−16***
1996	1.265e−01	6.013e−03	21.041	<2e−16***
1997	2.801e−01	5.757e−03	48.653	<2e−16***
1998	4.333e−01	5.715e−03	75.808	<2e−16***
1999	6.706e−01	5.558e−03	120.655	<2e−16***
2000	8.053e−01	5.452e−03	147.715	<2e−16***
2001	8.916e−01	5.325e−03	167.431	<2e−16***
2002	8.765e−01	5.241e−03	167.243	<2e−16***
2003	8.510e−01	5.216e−03	163.143	<2e−16***
2004	8.706e−01	5.204e−03	167.305	<2e−16***
2005	9.261e−01	5.067e−03	182.765	<2e−16***
2006	9.989e−01	5.020e−03	199.008	<2e−16***
2007	1.092e+00	4.996e−03	218.572	<2e−16***
2008	1.126e+00	5.004e−03	225.033	<2e−16***
2009	1.066e+00	5.085e−03	209.665	<2e−16***
2010	1.069e+00	5.116e−03	208.968	<2e−16***
2011	1.046e+00	5.116e−03	204.451	<2e−16***
2012	9.919e−01	5.148e−03	192.660	<2e−16***
2013	9.922e−01	5.222e−03	189.997	<2e−16***
2014	1.077e+00	5.136e−03	209.756	<2e−16***
M2	7.818e−03	2.517e−05	310.600	<2e−16***
NKAMERS	1.949e−02	7.371e−04	26.440	<2e−16***
AGE	−2.881e−03	3.135e−04	−9.190	<2e−16***
NVERDIEP	1.064e−02	1.223e−03	8.702	< 2e−16***
VERW1	−6.645e−02	3.651e−03	−18.203	<2e−16***
VERW2	5.185e−02	2.794e−03	18.559	<2e−16***
VERW3	1.019e−01	4.924e−02	2.070	0.038471*
ONBI2	1.059e−03	3.139e−02	0.034	0.973078
ONBI3	2.847e−02	1.483e−02	1.919	0.054992^.^
ONBI4	3.401e−02	1.772e−02	1.919	0.054934^.^
ONBI5	5.142e−02	1.411e−02	3.643	0.000269***
ONBI6	7.189e−02	1.452e−02	4.952	7.36e−07***
ONBI7	1.594e−01	1.396e−02	11.413	<2e−16***
ONBI8	2.596e−01	1.447e−02	17.946	<2e−16***
ONBI9	2.730e−01	1.403e−02	19.465	<2e−16***
Town house	9.764e−02	1.349e−02	7.236	4.65e−13***
Corner house	7.207e−02	4.735e−03	15.219	<2e−16***
Semi-detached house	2.391e−01	7.828e−03	30.545	<2e−16***
Detached house	2.633e−01	7.059e−03	37.293	<2e−16***
Apartment	−2.646e−02	2.912e−03	−9.087	<2e−16***
Loc36301	6.941e−02	3.442e−02	2.017	0.043736*
Loc36302	−2.047e−01	3.384e−03	−60.477	<2e−16***
Loc36303	−1.101e−01	3.096e−03	−35.572	<2e−16***
Loc36304	−2.625e−01	3.212e−03	−81.723	<2e−16***
Loc36305	−4.052e−01	3.640e−03	−111.322	<2e−16***
Loc36306	−2.735e−01	3.286e−03	−83.230	<2e−16***
Loc36307	−5.498e−01	3.174e−03	−173.192	<2e−16***
Loc36308	−5.697e−01	4.154e−03	−137.140	<2e−16***
Loc36309	−5.753e−01	3.623e−03	−158.809	<2e−16***
Loc36310	−4.531e−01	3.930e−03	−115.297	<2e−16***
Loc36311	−7.026e−01	3.456e−03	−203.322	<2e−16***
Loc36312	−2.098e−01	2.987e−03	−70.246	<2e−16***
Loc36313	−4.562e−02	2.398e−03	−19.022	<2e−16***
Loc36314	−1.973e−01	2.828e−03	−69.765	<2e−16***

1996−2014 are the year dummies, while 1995 is omitted for identifiability. Residual standard error: 0.2198 on 115235 degrees of freedom, Multiple R-squared: 0.8425, Adjusted R-squared: 0.8424, F-statistic: 1.163e+04 on 53 and 115235 DF, p value: < 2.2e−16. Signif. codes: 0.05 ‘$^.$’, 0.01 ‘*’, 0.001 ‘**’, 0 ‘***’

**Table 6:** Stationarity test for house price return series

**Series**	**ADF**		**KPSS**	
	Test statistics	*P* value	Test statistics	*P* value
CT	−2.42(1)	0.15	0.42(1)	0.07*
WP	−1.73(1)	0.40	0.56(1)	0.03**
OW	−2.25(1)	0.20	0.49(1)	0.04**
ZB	−1.92(1)	0.32	0.60(1)	0.02**
BL	−1.93(1)	0.31	0.58(1)	0.02***
DB	−1.83(1)	0.36	0.56(1)	0.03**
ND	−1.64(1)	0.44	0.75(1)	<0.01***
GS	−1.74(1)	0.39	0.78(1)	<0.01***
OD	−1.58(1)	0.47	0.70(1)	<0.01***
SO	−1.89(1)	0.33	0.58(1)	0.02**
ZO	−1.53(1)	0.49	0.82(1)	0.01***
WG	−2.02(1)	0.28	0.53(1)	0.04**
OZ	−2.12(1)	0.24	0.47(1)	0.05**
ZA	−1.99(1)	0.29	0.52(1)	0.04**

The test regression is estimated separately for each time series with an intercept. Due to the limited sample size, the augmented lag in the ADF procedure is set to one (indicated in the parenthesis). One indicated in parenthesis for the KPSS test is the Newey–West estimator of the bandwidth parameter. The null hypothesis for ADF is that the series contains unit root, while the KPSS null states that the series is stationary. *, ** and *** denote statistical significance at the 10, 5 and 1% respectively.

## Footnotes

1 By definition, the VaR is not sub-additive and thus not considered as a (coherent) risk measure. Heath et al. (1999) enumerates four axioms for which a metric must satisfy in order to be a coherent risk measure. Sub-additivity is one of these requirements and means the measure of risk of a portfolio must be less or equal to the sum of the risk measure of the individual assets that make up the portfolio.

2 For a real number *x*, the function $\min (x,0)$ equals *x*, if $x\,<\,0$ and 0 otherwise.

3 NVM is the Dutch National Association of Property Brokers. The association makes data available on request, following a number of strict procedures, and the sales data used in this paper were not directly accessible by the authors.

4 Properties with extremely small sizes (below 20 m$^2$) rarely exist in the Netherlands.

5 The population growth between 1990 and 2013 for Amsterdam was about 6.5% according to the CBS.

6 The lower observations in Westpoort are because the district is relatively new and the majority of the houses were built recently.
